# Photoacoustic Ophthalmoscopy: Principle, Application, and Future Directions

**DOI:** 10.3390/jimaging4120149

**Published:** 2018-12-12

**Authors:** Van Phuc Nguyen, Yannis M. Paulus

**Affiliations:** 1 Department of Ophthalmology and Visual Sciences, University of Michigan, Ann Arbor, MI 48105, USA; 2 Department of Biomedical Engineering, University of Michigan, Ann Arbor, MI 48105, USA

**Keywords:** photoacoustic ophthalmoscopy, photoacoustic microscopy, photoacoustic imaging, optical coherence tomography, multimodal imaging modality

## Abstract

Photoacoustic ophthalmoscopy (PAOM) is a novel, hybrid, non-ionizing, and non-invasive imaging technology that has been used to assess the retina. PAOM can provide both anatomic and functional retinal characterizations with high resolution, high sensitivity, high contrast, and a high depth of penetration. Thus, ocular diseases can be precisely detected and visualized at earlier stages, resulting in an improved understanding of pathophysiology, improved management, and the improved monitoring of retinal treatment to prevent vision loss. To better visualize ocular components such as retinal vessels, choroidal vessels, choroidal neovascularization, retinal neovascularization, and the retinal pigment epithelium, an advanced multimodal ocular imaging platform has been developed by a combination of PAOM with other optical imaging techniques such as optical coherence tomography (OCT), scanning laser ophthalmoscopy (SLO), and fluorescence microscopy. The multimodal images can be acquired from a single imaging system and co-registered on the same image plane, enabling an improved evaluation of disease. In this review, the potential application of photoacoustic ophthalmoscopy in both research and clinical diagnosis are discussed as a medical screening technique for the visualization of various ocular diseases. The basic principle and requirements of photoacoustic ocular imaging are introduced. Then, various photoacoustic microscopy imaging systems of the retina in animals are presented. Finally, the future development of PAOM and multimodal imaging is discussed.

## 1. Introduction

Vision impairment and blindness are a major public health problem that is increasing worldwide. According to the global prevalence of vision impairment and blindness, approximately 36 million people worldwide are blind [1]. In 2017, the prevalence of low vision and blindness for individuals over the age of 45 was estimated to be 3.9 million cases in the United States (USA) alone [2]. By 2030, an estimated 5.4 million people will be affected in the USA [2]. Importantly, more than 80% of such visual impairment is caused by retinal diseases such as age-related macular degeneration (AMD), glaucoma, and diabetic retinopathy [3]. Diabetic retinopathy is one of the leading causes of irreversible blindness in people over 50 years of age in the United States and the developed world. The prevalence rates for diabetic retinopathy and vision-threatening diabetic retinopathy are about 40.3% and 8.2%, respectively. In AMD, most visual loss occurs in the late stages of the disease, with a prevalence of 85–90% for wet AMD, and 10–15% for dry AMD. It is estimated that as much as $167 million could be saved annually with improved screening methods just for diabetic retinopathy [3]. Therefore, the earlier diagnosis and treatment of eye diseases are tremendously significant for healthcare.

Advanced ophthalmic imaging plays an important role in the monitoring and management of both healthy and abnormal ocular tissues. Advanced ophthalmic imaging can provide not only anatomy, but also functional information of the eye, enabling the diagnosis and detection of various diseases at an earlier stage. Several imaging modalities have classically been used to assess and evaluate the retinal and choroidal vasculature, including color fundus photography, fluorescein angiography (FA), indocyanine green angiography (ICGA) [4-6], optical coherence tomography (OCT), scanning laser ophthalmoscopy (SLO), fundus autofluorescence, fluorescent lifetime imaging ophthalmoscopy (FLIO), and optical coherence tomography angiography (OCTA) [4,7,8]. These imaging modalities depend on the detection of backscattering light from the retinal tissues or emitted light from an exogenous contrast agent, leading to limited penetration depth and visualization of the depth vasculature network. In contrast, photoacoustic (PA) ophthalmoscopy imaging is a new, emerging, and non-ionizing imaging technology. The major advantage of PA imaging is that optical contrast can be used to quantify both structural information (e.g., size, shape), and functional information (e.g., oxygenated and deoxygenated hemoglobin and oxygen saturation) [9-13]. Thus, PA imaging is highly desirable for imaging the eyes. Photoacoustic ophthalmoscopy (PAOM) imaging relies on the detection of the acoustic signal created by the absorption of short-pulsed laser light illuminated to reconstruct an image [14-21]. As a result, photoacoustic ophthalmoscopy enables the assessment of ocular tissues with high-resolution, high sensitivity, high-contrast, and a high depth of penetration, which is missing in most existing ophthalmic imaging systems.

Since the first introduction of photoacoustic ophthalmoscopy in 2010 to today, there has been a huge advance in photoacoustic ophthalmoscopy and its application in vivo imaging of the vasculature of the retina in rodents [22]. Recently, the integration of photoacoustic microscopy (PAM) with other imaging technologies such as OCT, FA, and adaptive optics (AO) have been developed to achieve multimodal imaging, which can significantly improve the image quality, temporal and spatial resolution, and increase the field of view to better visualize the targeted tissues in both 2D and 3D. The ocular images can be acquired from each imaging modality, and then these images can be co-registered, enabling the identification of very specific regions of interest based on anatomical structures and the photoacoustic signal. These remarkable advances in ophthalmic imaging can precisely help visualize and distinguish in detail retinal structures, their depth, and the surrounding anatomy better than conventional ophthalmic imaging systems. This review introduces the recent advances in photoacoustic ophthalmoscopy, as well as their basic principles, current applications, and future directions.

## 2. Physical Principle of Photoacoustic Imaging

**Figure 1.**
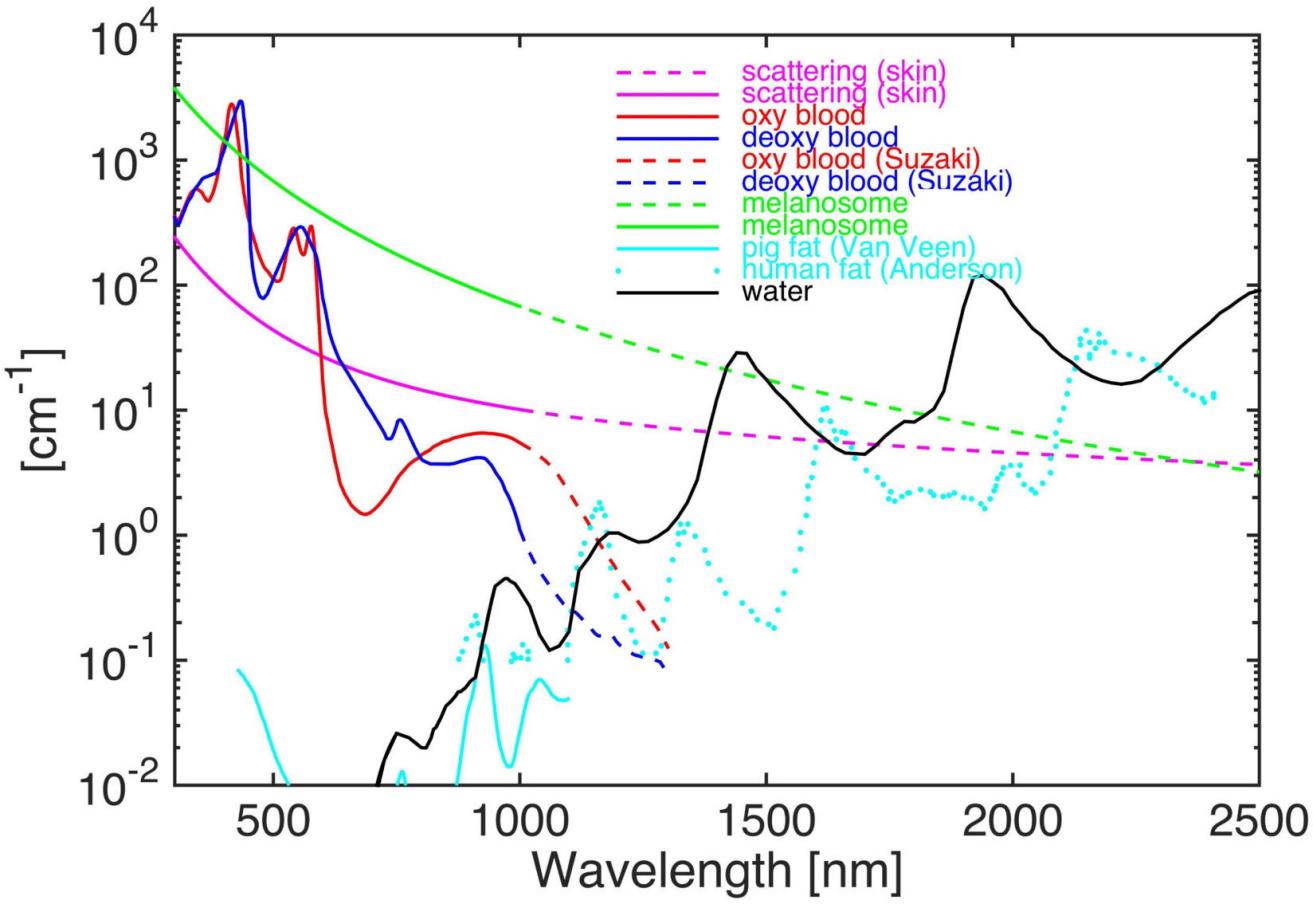
Absorption coefficient spectra of chromophores (water, oxygenated hemoglobin, deoxygenated hemoglobin, melanin, and fat) as a function of optical wavelengths [37]. Adapted from ref. [37].

**Figure 2.**
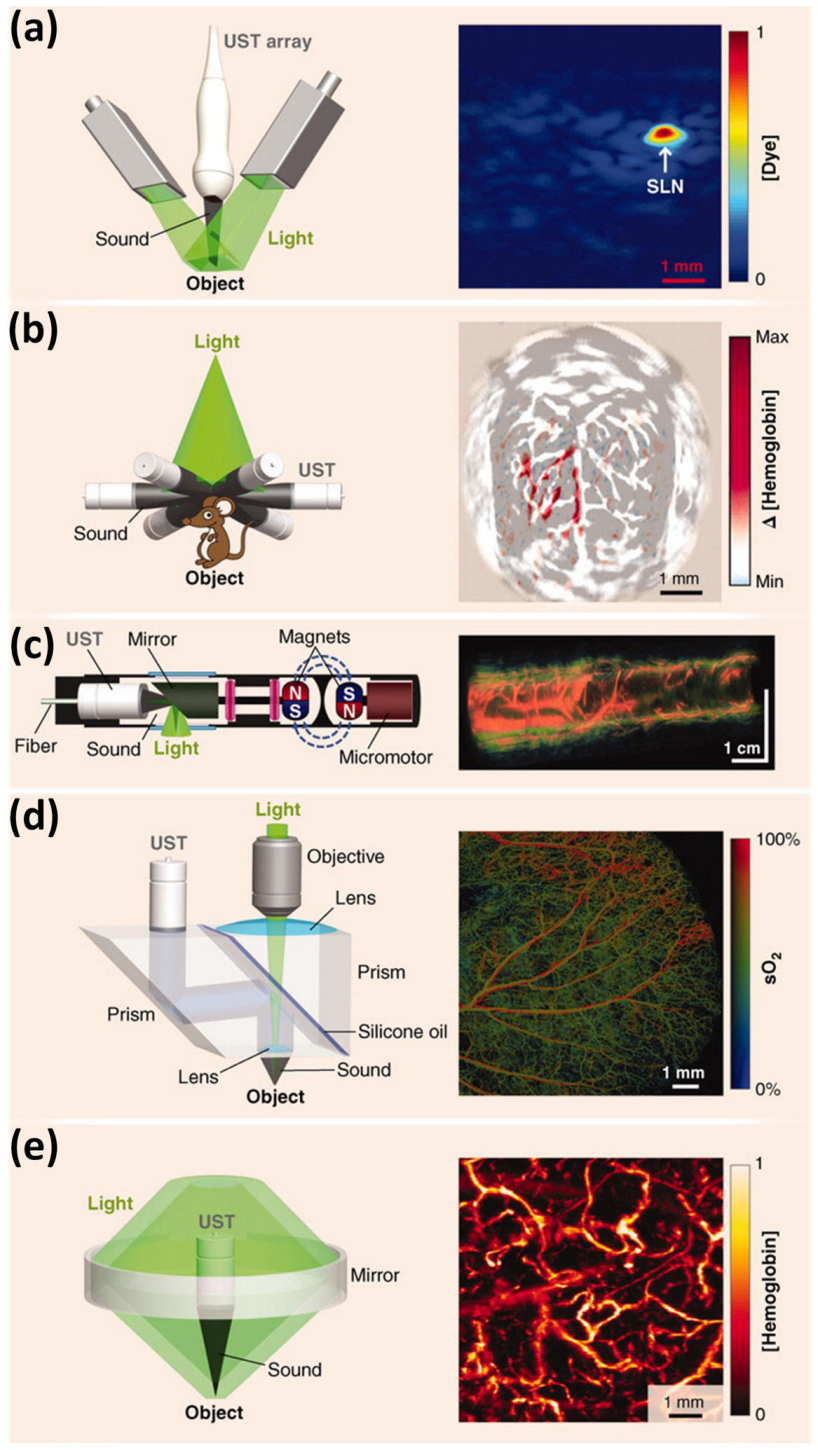
Photoacoustic (PA) imaging system and application in the medical field: (**a**) Linear-array photoacoustic tomography (PAT) of methylene blue concentration in a rat sentinel lymph node (SLN), (**b**) circular array PAT of cerebral hemodynamic changes in a rat, and (**c**) photoacoustic endoscopy (PAE) of a rabbit esophagus. UST: ultrasound transducer. (**d**) Optical resolution photoacoustic microscopy (OR-PAM) used for quantification of oxygen saturation in a mouse ear. (**e**) Acoustic resolution photoacoustic microscopy (AR-PAM) of normalized total hemoglobin concentration in a human palm [16]. Adapted with permission from ref. [16].

The physical principle of photoacoustic imaging (PAI) has widely been described by several groups [15-17,19,20,23]. In brief, short-pulsed laser light is used as the light source to illuminate the biological tissue being imaged. The biological tissue will absorb the deposited laser energy and convert it into heat, resulting in a rapid localized temperature increase and subsequent thermoelastic expansion. This expansion generates acoustic waves, which are termed photoacoustic waves. An ultrasound transducer is used to acquire the laser-induced acoustic signals. The transducer is placed in contact with the surface of the tissue or in contact with the conjunctiva of the eyes and aligned to enable an accurate alignment with the illuminating laser light. The recorded PA signal will be filtered and amplified using a low-noise amplifier. Finally, the signals are converted into digital signals and recorded using a high-speed digitizer to reconstruct two-dimensional (2D) or three-dimensional (3D) images. To maximize the creation of acoustic signals from the illuminating laser and enhance signal-to-noise ratio (SNR) of the PA images, the laser excitation time needs to be shorter than both the tissue’s thermal and stress relaxation time [24]. Laser pulses with a pulse width of three to 10 ns are usually used for PA imaging [16,18,24,25]. For image reconstruction, a single laser pulse excitation at a fixed position creates the acoustic signal, which is recorded and converted into a one-dimensional (1D) depth-resolved PA image along the Z-axis, which is referred to as an A-line. By implementing horizontal scanning lines along the X-axis of a sample, a two-dimensional depth-sensitive PA image is acquired. To obtain 3D volumetric PA images, each sample is scanned along the X and Y-directions using an optical scanning or mechanical scanning method. For quantitative evaluation, the PA amplitudes at the different regions of interest (ROIs) will be measured and compared. The PA amplitude depends on the optical absorption of the chromophores (i.e., melanin in the retinal pigment endothelium (RPE) or hemoglobin in blood), as shown in Figure 1. In addition, spectroscopic PA imaging is performed to determine the concentration of chromophore, or to quantify the suitable excited wavelength for distinguishing between normal and abnormal tissues [20,26]. Photoacoustic imaging is classified into three groups: photoacoustic tomography (PAT), photoacoustic microscopy (PAM), and photoacoustic endoscopy (PAE) (Figure 2) [15,16], [25,27,28]. PAT typically uses either a single or an array ultrasound transducer to detect the PA signal and target both microscopic and macroscopic imaging, whereas PAM and PAE usually use a focused ultrasound transducer to acquire PA signals and generally image tissue with micron-scale spatial resolution and millimeter-scale depth. The application of PA imaging has expanded from the cellular to the tissue level, such as imaging of the brain, blood vessels in the brain, liver, breast, joints, and blood vessels in the eye [17,29-31]. In ophthalmology, several groups have investigated PA ocular imaging platforms to evaluate ocular tissues with a high depth of penetration [18,22,32-35]. Due to high optical absorption properties within the visible light window (500–600 nm), PA imaging has the potential to precisely measure the concentration of oxygenated and deoxygenated hemoglobin in blood vessels and melanin. In addition, variations in retinal blood oxygen saturation (SO_2_) can be achieved [9,12,36]. Table 1 shows a brief summary of various ophthalmic imaging system, including PAT, PAOM, and versatile multimodality imaging platform (Vevo LAZR).

## 3. Requirement for Ocular Imaging

### 3.1. Safety Evaluation and Acquisition Speed

One of the major challenges of ocular imaging regards rapidly achieving high-resolution images without damaging sensitive neural tissue. Therefore, the ocular imaging system must meet several requirements. (1) Light safety plays a critical role in ocular imaging. The illumination intensity light energy that is used for ocular imaging must be below the American National Standards Institute (ANSI) safety limit standard. Delori et al. and Organisciak et al. have demonstrated that the retina can be injured by higher intensity exposure, leading to thermal damage, thermoacoustic damage, and photochemical damage [45,46]. The calculation of PA laser exposure has been described by Chao et al. [42]. In brief, ocular laser safety exposure dose depends on several parameters such as wavelength, exposure duration, illumination beam size, pulse repetition rate, and the average focal length of the eye. ANSI has three different rules: single pulse limit, the average power limit, and the repetitive pulse limit to regulate the maximum permissible exposure (MPE). The ANSI MPE limits for retinal exposure to nanosecond pulses in the 400 nm to 700 nm spectral range is 5.0 × 10^−7^J.cm^−2^ [42,47,48].

For a single-pulse MPE calculation, assuming a beam diameter matched to a fully dilated pupil (six mm): Gaussian 1/e^2^ diameter: D_64_ = 3.5 mm (64% of energy; ANSI convention)

Fluence on the cornea:

(1) $$\Gamma =\frac{E}{A}=\frac{0.64E_{p}}{\mathrm{PI}{\left(\frac{D_{64}}{2}\right)}^{2}}$$

(2) $$\Gamma =6.65\;E_{p}\;J.{\mathrm{cm}}^{-2}(\text{where}\;E_{p}\;\text{is energy in joule})$$

(3) $$\Gamma <\text{MPE}=5.0\;10^{-7}\;J.{\mathrm{cm}}^{-2}\;\text{for nanosecond visible light}$$

Thus, the max energy for single pulse exposure is:

(4) $$E=\frac{\Gamma }{6.65}=75\;\text{nJ}$$

(2) High imaging speed is needed to avoid possible motion artifacts. Robinson et al. have reported that the eye has a fixation time of approximately 500 ms [49]. This motion can cause image blurring or image disruption. (3) Non-invasive or minimally invasive imaging is highly desirable to reduce systemic risk and side effects such as nausea, vomiting, allergic reactions, and patient discomfort by the administration of exogenous contrast agents.

### 3.2. Photoacoustic Quantification

#### 3.2.1. Photoacoustic Amplitudes

The PA amplitude can be determined by using the following formula [21]:

(5) $$p_{0}=\Gamma \;\times \mu_{\mathrm{Hb}}\;\times \Phi$$

where Γ represents the Grüneisen coefficient, μ_Hb_ is the optical absorption coefficient of hemoglobin (cm^−1^), and Φ is the laser irradiance (W/cm^2^). Please note that this equation only predicts the PA signal at the absorber. During in vivo experiments, the incident laser light and the resulting PA signal will be attenuated in tissue (i.e., optical and acoustic scattering and absorption).

In the case of using the exogenous contrast agents, laser light propagates into the biological tissues such as blood vessels, and will be absorbed by both the nanoparticle and hemoglobin. Thus, the PA amplitude is proportional to the total optical absorption coefficient of the photoabsorber (μ_a_ (cm^−1^)), and the optical absorption coefficient of hemoglobin (μ_Hb_ (cm^−1^)). Thus, Equation (5) can be rewritten as follows:

(6) $$p_{0}=\Gamma \;\mu_{t}\;\Phi$$

where:

(7) $$\mu_{t}=\mu_{a}+\mu_{\mathrm{Hb}}$$

#### 3.2.2. Photoacoustic Contrast

Photoacoustic image contrast is defined as the fractional variation in the average photoacoustic amplitudes extracted from different regions of interest (ROI). The PA image contrast is determined as follows [17,21]:

(8) $$\text{Contrast}=\frac{{\bar{\mathrm{PA}}}_{T}-{\bar{\mathrm{PA}}}_{\mathrm{BG}}}{{\bar{\mathrm{PA}}}_{\mathrm{BG}}}$$

where ${\bar{\mathrm{PA}}}_{T}$ and ${\bar{\mathrm{PA}}}_{\mathrm{BG}}$ are the average PA amplitudes from the targeted tissues and adjacent background, respectively.

#### 3.2.3. Oxygen Saturation (SO_2_)

The amplitude of the photoacoustic signal is proportional to the local optical absorption coefficient. Thus, the relative oxygenated-hemoglobin (HbO_2_) and deoxygenated hemoglobin (Hb) concentration can be estimated as follows [35]:

(9) $$\mu_{a}(\lambda_{1})=2.303\times (E_{HbO_{2}}(\lambda_{1})C_{HhO_{2}}+E_{Hh}(\lambda_{1})C_{Hb})$$

(10) $$\mu_{a}(\lambda_{2})=2.303\times (E_{HbO_{2}}(\lambda_{2})C_{HhO_{2}}+E_{Hh}(\lambda_{2})C_{Hb})$$

where *μ*_a_(*λ*_1_) and *μ*_*a*_ (*λ*_2_) are the absorption coefficients of the blood at selected wavelengths *λ*_1_ and *λ*_2_. E and C represent the molar extinction coefficients and concentration, respectively. HbO_2_, and Hb denote the oxygenated hemoglobin and deoxygenated hemoglobin, respectively.

The oxygen saturation of hemoglobin can be obtained as follows:

(11) $${\mathrm{SO}}_{2}=\frac{C_{HhO_{2}}}{C_{HhO_{2}}+C_{Hb}}$$

### 3.3. Imaging Resolution of Photoacoustic Ophthalmoscopy

Recently, PAM has been investigated as a non-invasive technique for the detection of the vasculature of the eye [22,25,42]. PAM imaging can be divided into two groups: acoustic resolution PAM (AR-PAM) and optical resolution PAM (OR-PAM) [36,50,51]. Figure 2d,e depicts schematic diagrams of AR-PAM and OR-PAM. For AR-PAM, a diffusing laser light is used to illuminate the tissues, and the induced PA signals are detected by a focused ultrasound transducer. The focus beam of an ultrasound transducer determines the lateral resolution of AR-PAM, whereas the axial resolution is determined by the ultrasound frequency and bandwidth [21,24,52,53]. The lateral resolution of AR-PAM is determined using the following formula [54]:

(12) $$\mathrm{LR}=\frac{0.71\;\lambda }{{\mathrm{NA}}_{a}}$$

where LR denotes the lateral resolution, *λ* is the acoustic wavelength, and NA_*a*_ is the numerical aperture of the focused ultrasound transducer.

In OR-PAM, both the excitation light and ultrasound detection are tightly focused onto the ocular tissue and co-registered in the confocal plane. The optical focal beam that is used to generate the PA signal determines the lateral resolution. The lateral resolution of OR-PAM is given by [54]:

(13) $${\mathrm{LR}}_{o}=\frac{0.71\;\lambda_{o}}{{\mathrm{NA}}_{o}}$$

where LR_*o*_ denotes the lateral resolution of OR-PAM, *λ_o_* is the optical wavelength and NA_*o*_ is the numerical aperture (NA) of the optical objective.

However, the axial resolution is still determined by the ultrasound transducer parameters [55]. The axial resolutions of both OR-PAM and AR-PAM are determined by the bandwidth of the ultrasound transducer. The axial resolutions are estimated using the following formula [54]:

(14) $$\mathrm{AR}=\frac{0.88c}{\text{bandwidth}}$$

where AR represents the axial resolution (μm), and c is the speed of sound in medium (c = 1.54 mm/μs).

Unlike any of the current variants of optical microscopy, an important distinguishing feature of OR-PAM is that it provides an optical absorption-based image contrast. However, most optical microscopy has a limited penetration depth of approximately one millimeter in tissues due to strong scattering. The lateral and axial resolution in OR-PAM are summarized in Table 2 as the following:

Compared with AR-PAM, OR-PAM provides much higher lateral resolution. Thus, OR-PAM system can be used to image individual capillaries in vivo with high contrast [25,42,51,55].

### 3.4. Scanning Mode of Photoacoustic Ophthalmoscopy

#### 3.4.1. Mechanical Scanning

**Figure 3.**
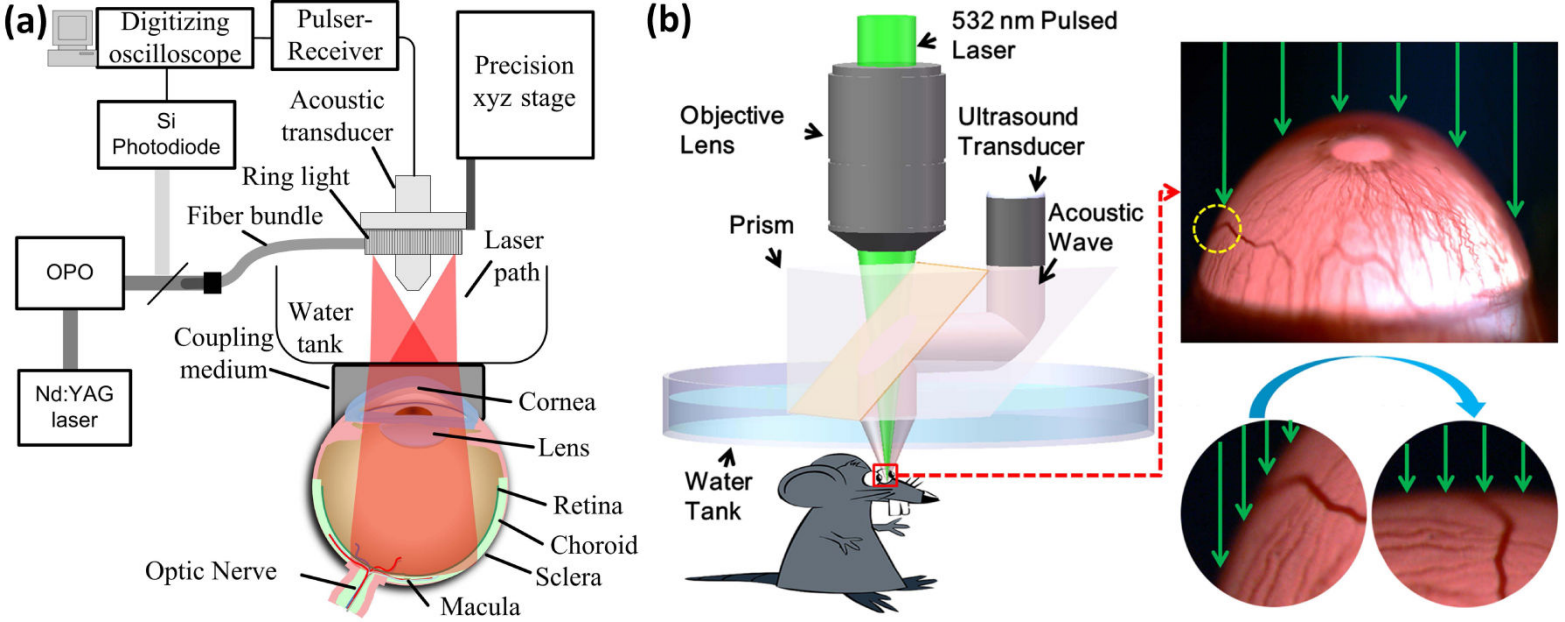
The PAM system used for imaging the eye in living rabbit: (**a**) schematic diagram of the acoustic resolution photoacoustic microscopy (AR-PAM). Laser excitation wavelength of 740 nm. (**b**) Diagram of the optical resolution photoacoustic microscopy (OR-PAM). Laser excitation wavelength of 532 nm. Adapted with permission from ref. [22].

To acquire 3D volumetric imaging of ocular tissue, both AR-PAM, and OR-PAM perform raster-scanning along the *X* and *Y*-axes mechanically or optically. Therefore, a PA imaging system can also be classified into mechanical scanning and optical scanning imaging systems. In the mechanical scanning mode, both the optical excitation and ultrasound transducer are simultaneously scanned over a planar surface, generating and detecting a PA signal at each step of the scan. In addition, a water tank is required to maintain ultrasound coupling during imaging [16,22]. An example reported by de la Zerda et al. shows a PAM image of a living rabbit eye obtained using an AR-PAM mechanical scanning technique to visualize eye tissues, including the iris, cornea, lens, retina, choroid, sclera, and blood vessels [22]. The experiment setup is exhibited in Figure 3a. To couple the acoustic signal between the tissue and ultrasound detector, a water tank was used and mounted on the surface of the rabbit eye. The optical wavelength of 740 nm was used to illuminate the eye tissue to avoid the light absorption of melanin. To detect the PA signal, an ultrasound transducer with a center frequency of 15 MHz was applied. However, it is difficult to evaluate retinal microvasculature from the maximum intensity projection (MIP) PA image due to limited ultrasound spatial resolution (approximately 200 μm). Furthermore, the acquisition time is approximately 90 minutes to acquire the volumetric visualization of the PA signal. Figure 3b shows a diagram of OR-PAM. The acoustic signal was generated by the excitation wavelength of 532 nm.

#### 3.4.2. Optical Scanning

In the optical scanning mode, the ultrasound transducer is kept stationary, while the focused optical illumination is raster-scanned using galvanometers [25,33,57]. The advantages of optical scanning are that it can provide higher scanning speed in comparison with mechanical scanning. Importantly, optical scanning is suitable for retinal imaging and compatibility with optical coherence tomography (OCT) and SLO [24,57]

**Figure 4.**
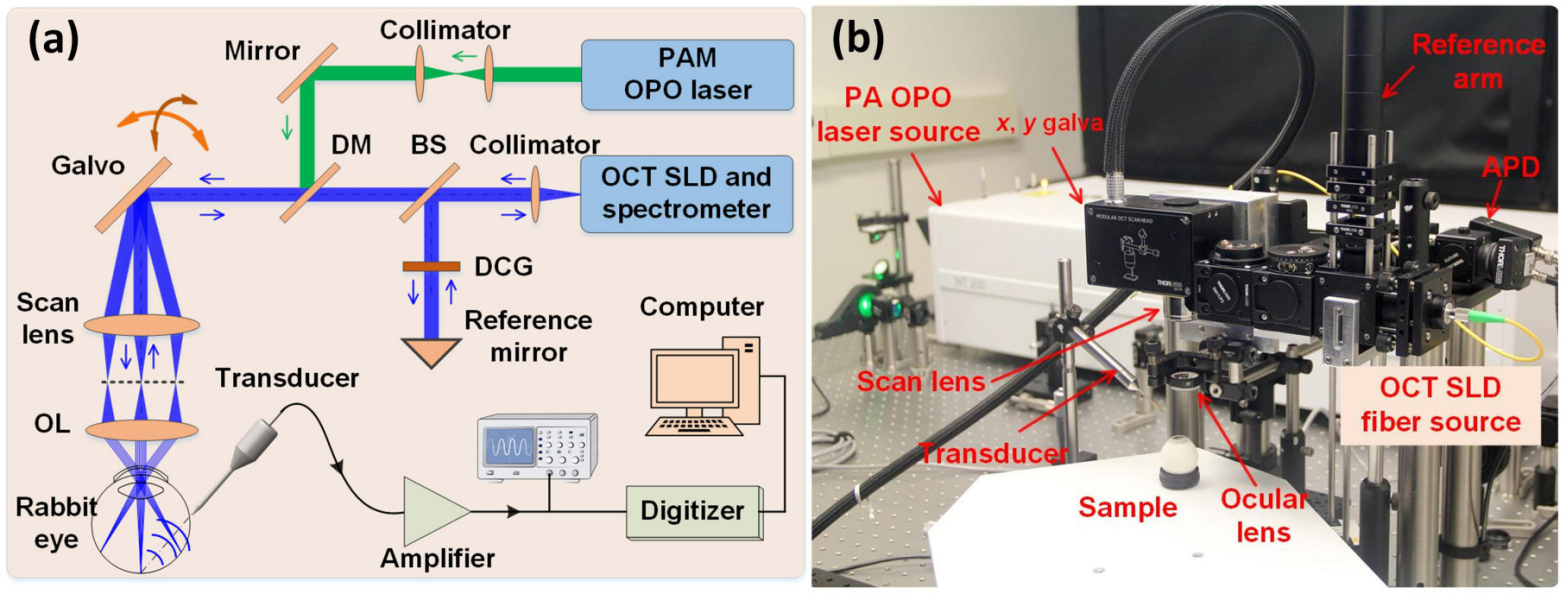
An optical scanning photoacoustic microscopy system for the imaging of retinal and choroidal blood vessels: (**a**) schematic diagram of optical scanning showing the ultrasound transducer position and the illumination light enters the eye and focuses on the retina, and (**b**) physical setup [14]. Adapted with permission from ref. [43].

Tan et al. developed an optical-scanning PAM system, termed photoacoustic ophthalmoscopy (PAOM) to measure the optical absorption properties of the retina [58]. Figure 4a illustrates the schematic diagram of their system. Figure 4b shows the physical setup. The authors used a 570-nm laser pulse as a light source to generate the PA waves. The laser light was delivered through a single mode fiber and then collimated before threading through a pair of galvanometer mirrors. The laser beam size was adjusted to be two millimeters on the pupil plane. Laser-induced PA signals were detected by a custom-built unfocused needle ultrasonic transducer with a central frequency of 27 MHz, 16 MHz bandwidth, and field of view of 4 × 4 mm^2^. The ultrasound transducer was gently placed in contact with the eyelid and coupled with ultrasound gel. The lateral and axial resolution of the system were 37.0 μm and 4.1 μm, respectively.

## 4. Contrast Agents

Although the vasculature in the retina and choroid can be imaged using PAM without using a contrast agent, the diagnostic information is limited to hemodynamic properties such as blood flow, blood volume, and oxygen saturation. The sensitivity and specificity of PAM can be improved by adding exogenous contrast agents as shown in Table 3, and the application scope of PAM can be extended from the tissue level to the molecular and cellular levels. Exogenous contrast agents for PA imaging can be classified into two groups: organic (e.g., liposomes, dyes, indocyanine green (ICG), fluorescein sodium, Prussian blue, methylene blue, and polymeric complexes), and inorganic (e.g., gold nanoparticles, silica [59,60], and copper sulfide nanoparticles [61,62]). Each agent has advantages and limitations. For example, ICG is a dye approved by the Federal Drug Administration (FDA) for clinical use. ICG has high optical absorption in the near-infrared (NIR) spectral window. Additionally, ICG has already been shown to increase the PA signal of blood vessels after intravenous administration [23]. Although organic dyes are non-toxic and have a more established route toward clinical translation, without conjugating to other chemicals, they are easily biodegradable, often ropidly cleared from the body, and often have a lower PA signal. Additionally, the molecule of fluorescein is highly fluorescent (QY = 079), resulting in creating a weak PA signal. Thus, they are of limited application in long-term PA monitoring. Yoo et al. have demonstrated that the use of Cy7 fluorophore allowed enhancing OCT image contrast, leading; to improving the visualization of artery and vessel walls [63]. However, the level of contrast enhancement by organic agents can be limited. Compared with organic dyes, inorganic contrast agents such as gold particles (AuNPs) and the quantum dot have exhibited promising results in photoacoustic molecular imaging [17]. Recently, Hu et al. discovered the quantum dot for both OCT and PAM with promising results in the detection of blood vessels in vivo [64]. The use of AuNPs for OCT has also been studied on cells by using gold nanoshells as the contrast agent to enhance OCT signals [65]. As a result of the surface plasmon resonance, AuNPs have very unique optiral properties, including extremely strong optical aOsorption and optical scattering, making them excellent candidates as multimodal contrast agents for many optical imaging modalitiea. AuNPs also have excellent biostability, photostability, thermal stability, and optical tunability. By changing the size and shape, the optical absorption peak of AuNPs can be tuned throughout the visible and near-infrared region [19,66-69]. de la Zerda et al. have reported that large gold nanorods (LGNRs) (~100 × 30 nm) could enhance an OCT spectral signal approximately 110 times greater per particle than conventional gold nanorods (GNRs) (~50 × 15 nm) [70]. They also reported that nanorods were not toxic at the injection dose of nanoparticles. However, long-term toxicity needs to be examined. In addition, the chemical synthesis of AuNP can have associated toxicities [71-74]. Importantly, the local accumulation of targeted contrast agents that are used in multimodal PAM and OCT imaging could be quantified by performing spectroscopic PA image. Spectroscopic PAM can provide not only oxygen saturation estimation, but also information about disease processes at a cellular or molecular level.

The majority of the exogenous contrast agents that are used for PAM and OCT imaging have absorption spectrums in the NIR window (700–900 nm). Using these frequencies reduces scattering by the tissue, and avoids absorption by endogenous chromophores. More than 20 NIR contrast agents (free, bound to other chemicals, or conjugated with monoclonal antibodies) have been used in clinical trials such as FLARE (Fluorescence-Assisted Resection and Exploration) [75], NIR goggles [76], Spy Elite [77], and da Vinci [78]. Despite the notable progress employing the NIR window, the major disadvantage of shallow tissue penetration has remained the limiting factor, which restricts application to access tissues in humans. Thus, deeper tissue penetration requires using wavelengths longer than 900 nm. Recently, a second NIR window (900–1800 nm) and shortwave infrared radiation (SWIR) have been investigated for preclinical studies in larger animals such as rabbits, dogs, and pigs. When compared to the traditional NIR ranges, the second NIR window and SWIR demonstrate a higher tissue penetrance and transparency, with some of the most prominent bands of transparency at 1300 nm and 1550 nm [79].

## 5. Specific Application of Photoacoustic Microscopy for Imaging of Retinal Diseases in Rodents

### 5.1. Anterior PA Imaging of the Eye

**Figure 5.**
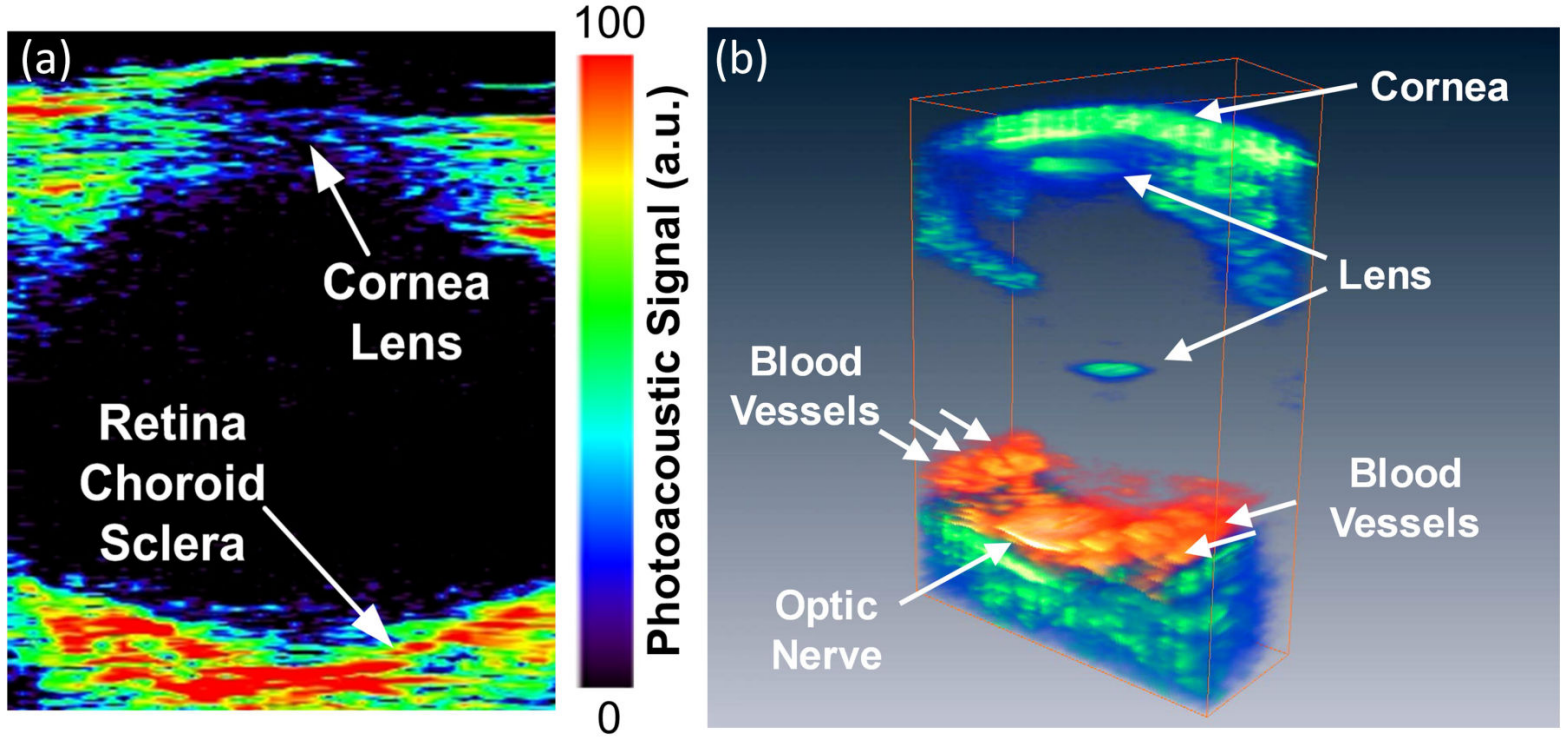
(**a**) Maximum intensity projection (MIP) PAM image (FOV = 12 × 8 mm) of the rabbit eye. The PAM image shows different types of retinal tissues such as, retinal choroid sclera, cornea lens, blood vessels, and optics nerve, and (**b**) three-dimensional (3D) visualization of the photoacoustic signal from the posterior eye. Laser excitation wavelength of 740 nm. Adapted with permission from ref. [22].

de la Zerda et al. has successfully applied PA imaging to image the retinal vessels [22]. The eye was imaged, which is displayed in Figure 5, illustrating the location of ocular components such as retinal blood vessels, iris, and cornea.

### 5.2. Retinal, Choroidal Vessels, Iris, Limbal Blood Vessels, and RPE

**Figure 6.**
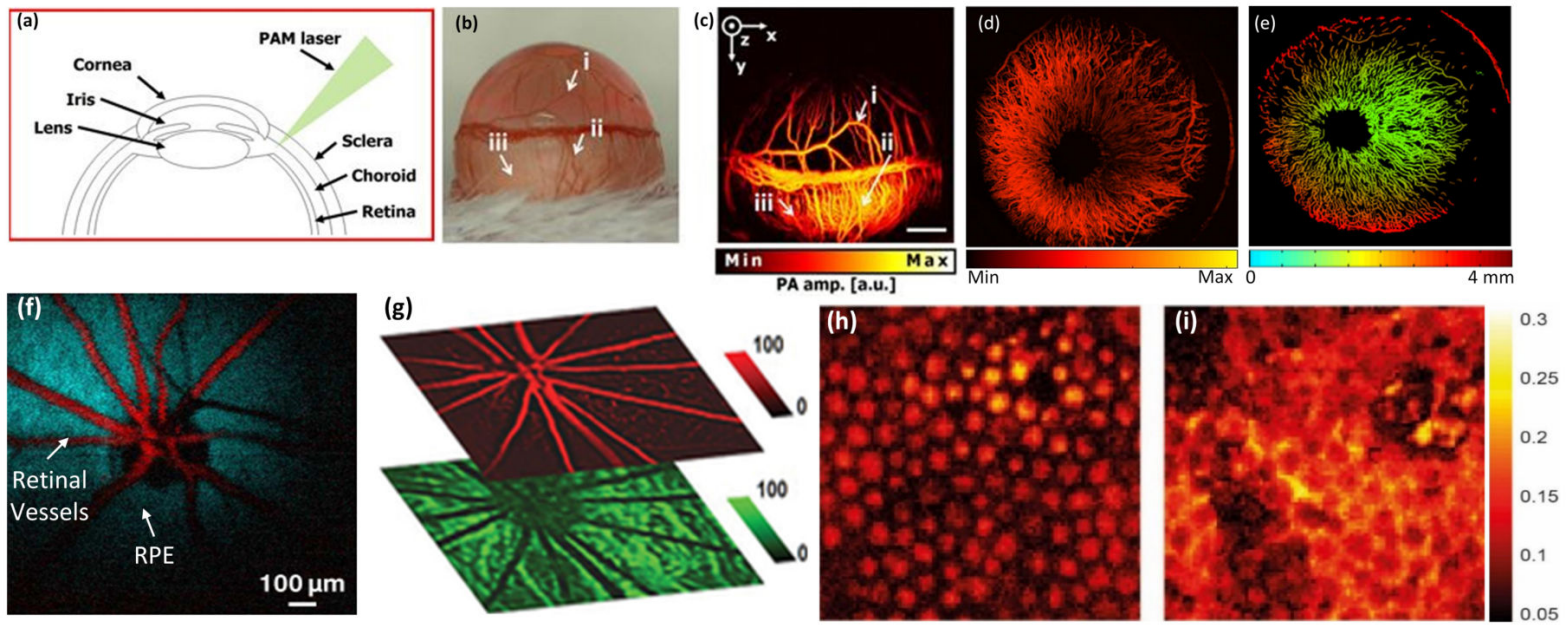
Photoacoustic microscopy of the retinal: (**a**) structure of mouse eye, (**b**) a photograph of the mouse eye, (**c**) corresponding MIP PAM image of the corneal microvasculature in an albino mouse eye in (**b**), showing the iris, and limbal blood vessels as well as the retinal and choroidal vessels underlying the sclera [51]. (**d**) Photoacoustic microscopy of the iris vasculature of rat. (**e**) Depth-encoded PAM image [83]. (**f**) Pseudo-colored PAOM images of the retinal vessels and RPE [33]. (**g**) Segmented PAOM of the retinal and choroidal blood vessels. The retinal vessels are pseudo-colored in red, and the choroidal vasculatures are pseudo-colored in green [58]. (**h,i**) segmented PAM porcine retinal pigment endothelium (RPE) and choroid [84]. Adapted with permission from ref [33,51,83,84].

OR-PAM have been developed to visualize the structural information of the microvascular network in the eye? [36,51], as shown in Figure 6. Joen et al. have conducted in vivo imaging to evaluate blood circulation and obtain high-resolution in vivo images of the eye using; OR-PAM (Figure 6a-c). Figure 6c shows an image of the eye microvasculature obtained using OR-PAM illustrating the iris and limbal blood vessels, as well as choroidal and retinal vasculatures underlying the sclera. In order to evaluate multiple layers and the curved structures of the eye, an ocular surface estimation algorithm based on a machine learning method, termed a random sample consensus algorithm (RANSAC), was applied. This resulted in the isolation of supra-surface vessels and surface vessels. As shown in Figure 4, individual retinal microvasculature was visualized, where the vessels diameters were estimated to be six μm.

Another study reported by Zhao et al. utilizes OR-PAM to detect and evaluate single iris vessels [83] (Figure 6d,e). In that study, they used a 75-MHz transducer to record the PA signal, and the excitation laser light was tightly focused onto the iris. The lateral resolution was approximately five μm. Individual capillaries were imaged with high contrast and high resolution, where the diameter was identified to be six μm. Several groups have successfully applied PAOM imaging platforms to evaluate ocular tissues, including; retinal vasculature, choroidal vasculature, and RPE melanin with a high depth of penetration [18,22,32-35], as shown in Figure 6f-i.

### 5.3. Corneal Neovascularization

**Figure 7.**
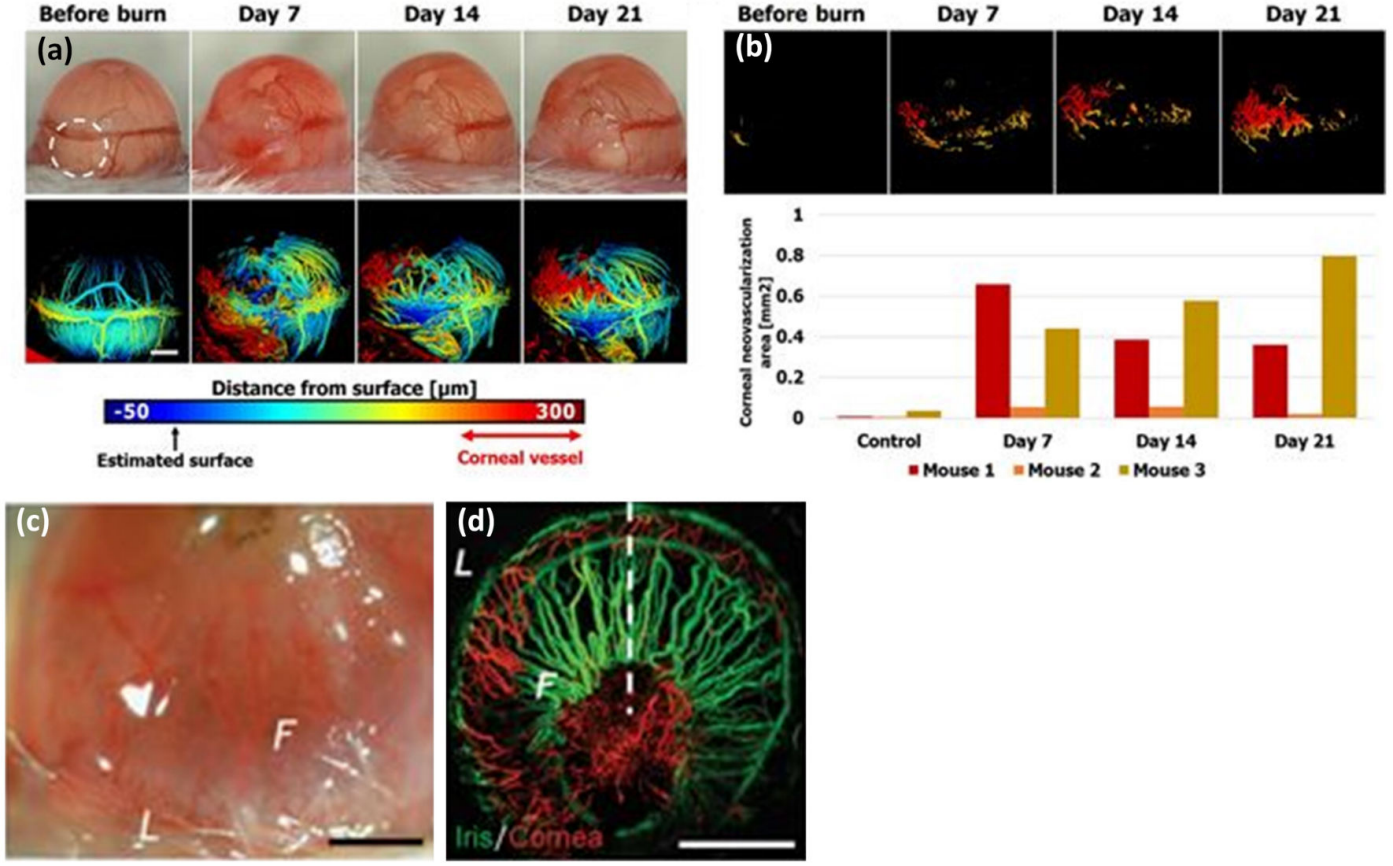
PAOM of corneal neovascularization. (**a**) Surface-based depth encoded PAM images of corneal before and after alkali burn-induced corneal neovascularization at various times. (**b**) Segmented PAM image of supra-surface vessels and quantified corneal neovascularization area [51], (**c**) bright field microscopy image of the eye, and (**d**) PAM distinguishes between the iris (green) and corneal (red) vasculatures, due to their different depths. The green arrow points toward iris vessels; the red arrow points toward corneal neovascularization in the Z-slice. F: front, and L: limbus [85]. Adapted with permission from ref. [51,85].

Joen and Kelly-Goss have successfully applied PAOM to detect corneal vascularization induced by alkali burns (Figure 7) [51,85].

### 5.4. Choroidal Neovascularization

**Figure 8.**
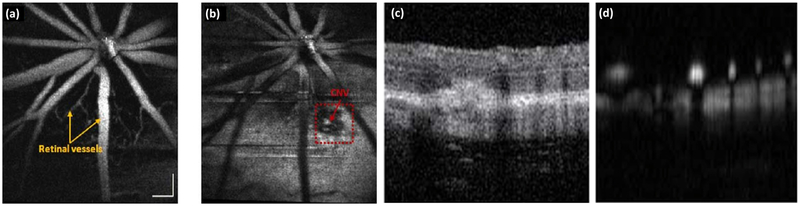
PAOM of choroidal neovascularization in mice. (**a**) Two-dimensional (2D) enface image of anterior retinal structure. (**b**) PAM image of the laser-induced CNV regions. Red arrows point to the CNV region. (**c**) and (**d**) are OCT B-Scan and PAM B-Scan, respectively. CNV: choroidal neovascularization Bar: 100 μm. Reproduced with permission from ref. [86].

In order to image choroidal neovascularization, the resolution of the PAOM imaging system required at least a submicron spatial resolution using focused light and sound to achieve the high resolution image of capillaries that were 10 μm in diameter. Thus, OR-PAM is usually utilized for the detection of capillaries. Dai et al. obtained dual PAM and OCT images of laser-induced choroidal neovascularization (CNV) in mice (Figure 8) [86]. CNV was induced by the illumination of laser light at high power. The reed dotted square box shows the position of the developed CNV obtained by the PAM system.

### 5.5. Retinal and Choroidal PAM Imaging in Larger Animal

To better visualize individual retinal vessels for retinal disease diagnosis, the PAOM imaging platform can be combined with other imaging modalities to address these requirements and achieve multimodal retinal imaging. Multimodal imaging provides several advantages by combining different contrast mechanisms to obtain not only comprehensive structural information, but also functional information.

**Figure 9.**
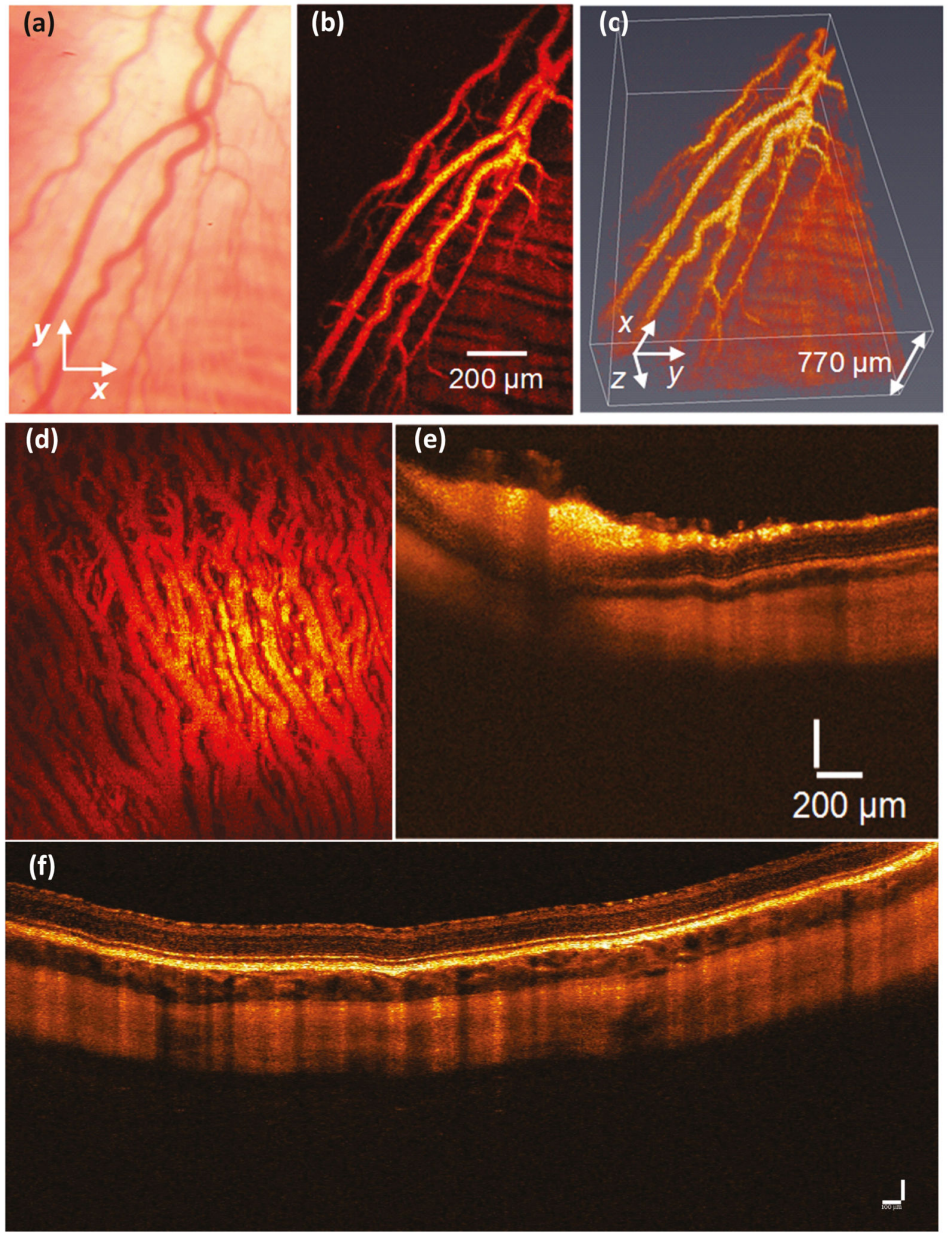
Photoacoustic microscopy of retinal and choroidal blood vessels in living rabbits: (**a**) color fundus photography, (**b**) corresponding PAM of retinal blood vessels, (**c**) 3D volumetric visualization of PA signal, (**d**) MIP PAM of choroidal vessels, (**e,f**) B-scan OCT images of retinal and choroidal blood vessels [14]. Adapted with permission from ref. [43].

Combining PAOM with autofluorescence imaging can potentially provide information on the retinal vasculature and the distribution and concentration of retinal melanin and lipofuscin, retinal blood flow, and retinal oxygen saturation SO_2_ [57,87]. Liu, Jiao, and Song et al. have described an integrated PAOM and OCT to visualize retinal vessels, choroidal vessels, and the RPE [33,50,88]. The combination of PAOM and OCT has several advantages. First, multimodal imaging can be achieved simultaneously from a single imaging system; then, the acquired images can be coherently registered and displayed on a single image plane. Second, the retinal vessels can be monitored in real-time with OCT. Third, the OCT system could help quantify single retinal capillaries and distinguish different layers of the retina, choroid, and sclera to supplement the information provided by PAM. Fourth, during. an in vivo experiment, OCT can be used as an alignment tool to guide PAOM. However, a limitation of these studies is that the eyeball of the small animal is much smaller than human eyes (e.g., ~three mm for mice, ~six mm for rats versus ~23 mm for human), resulting in a difficulty for translation to clinical applications, particularly for the ultrasound portion of PAM. Recently, our group has successfully developed a multimodal PAOM and OCT imaging platform that allows the visualization of both retinal and choroidal vasculature in living rabbits with high temporal and spatial resolution [89]. The advantage of our multimodal imaging platform is that the eyeball of the rabbit is similar to the human eyeball axial length (~18 mm for rabbits), which is an important step for studies of retinal diseases with PAM and translations to clinical application. Figure 9 shows PAM and OCT obtained in vivo in rabbit retinal and choroidal vessels. In order to achieve the high contrast of blood vessels, the PA images were obtained at a peak absorption of hemoglobin at the wavelength of 570 nm [90].

### 5.6. Laser-Induced Burn in Choroidal Vessels

**Figure 10.**
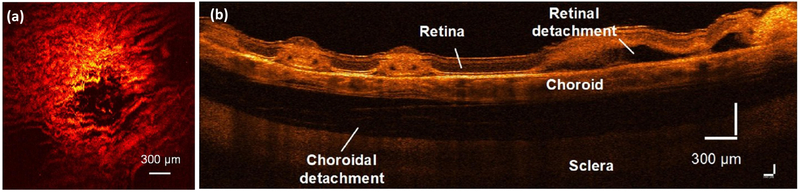
Multimodal photoacoustic microscopy (PAM), and optical coherence tomography (OCT) for chorioretinal imaging. (**a**) MIP PAM of laser-induced burn in choroidal vessels. (**b**) B-scan OCT image showing the location of laser treatment as well as retinal detachment [39]. Adapted with permission from ref. [39].

A further development in PAOM involves the combination of triple state-of-art imaging modalities including PAM, OCT, and fluorescein microscopy (FM) [39]. This multimodal imaging system provides excellent performance and can visualize not only retinal vessels, but also detect the change in vessels such as laser-induced choroidal neovascularization and retinal and choroidal detachment, as shown in Figure 10.

### 5.7. SO_2_ Measurements

**Figure 11.**
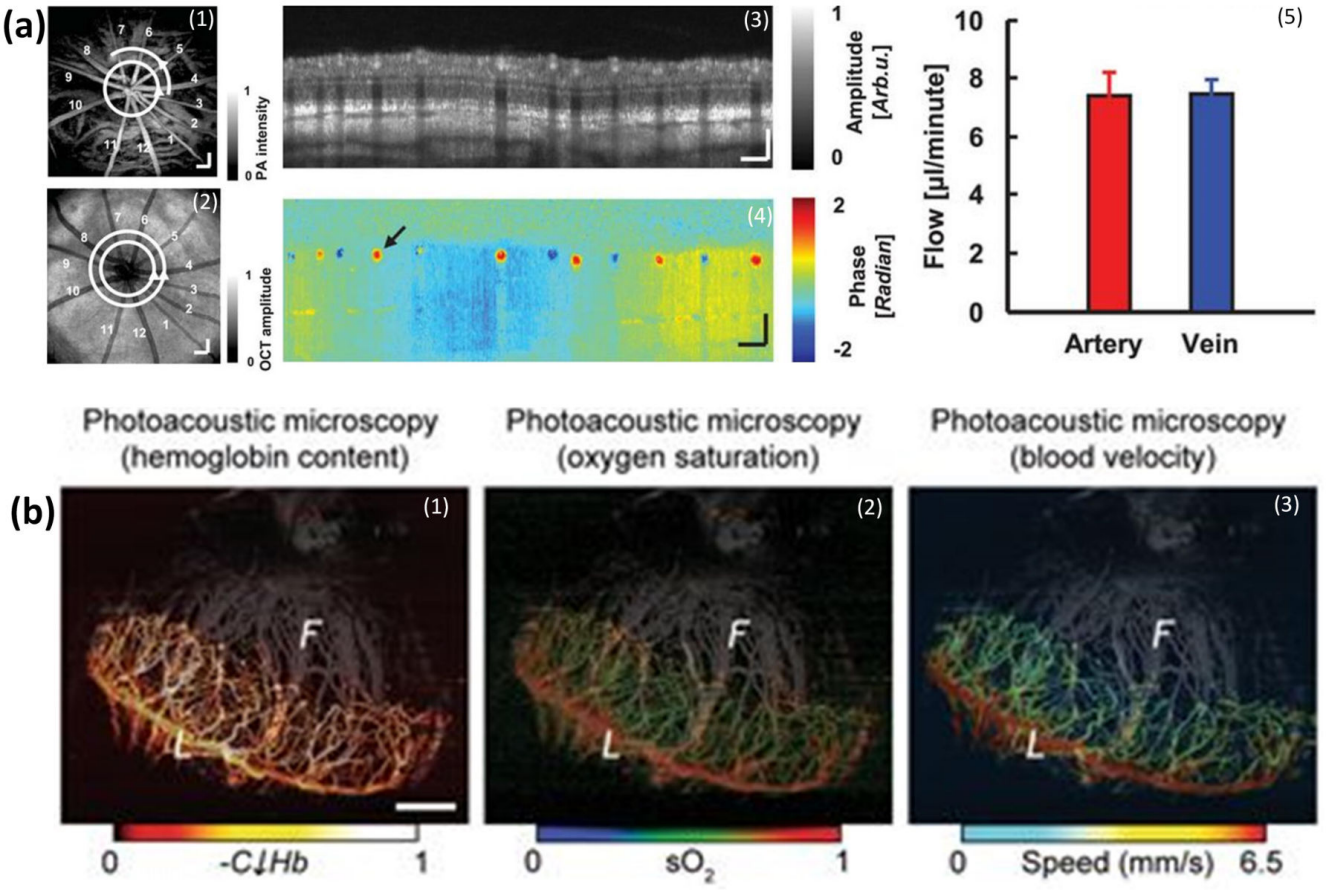
(**a**) Integrated PAOM and OCT for the quantification of a retinal oxygen metabolic rate. (a1) shows an en face PAM image of the retinal and choroidal vessels obtained at the wavelength of 570 nm. (a2) En face OCT image, (a3) B-scan OCT image acquired along the white circle in (a1), (a4) phase shift OCT image, and (a5) measured flow rate in the retinal and venous systems [9]. (**b**) Functional imaging of relative hemoglobin content (“CHb”), SO_2_ saturation, and blood velocity in the angiogenic corneal network obtained using PAOM. The iris vasculature, which is pseudo-colored in gray based on vessel depth, is visible in the background of each image [85]. Adapted with permission from [9,85].

**Figure 12.**
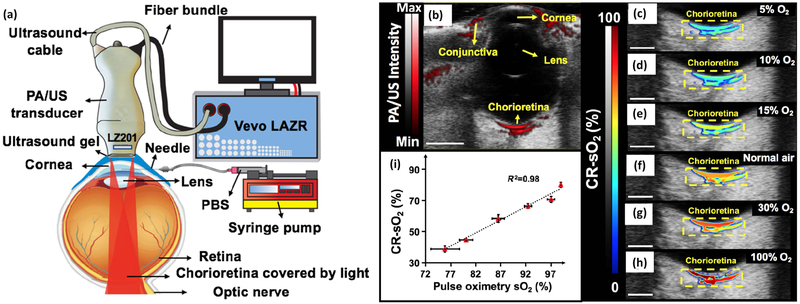
Ultrasound-resolution photoacoustic microscopy imaging for the detection of choroidal and retinal oxygen saturation (CR-SO_2_). (**a**) The ischemia-reperfusion setup. (**b–h**) Evaluation of the PAOM response to oxygen tensions. (**b**) B-mode photoacoustic/ultrasound image at baseline condition (breathing normal air) including the cornea, conjunctiva, lens, and chorioretina. The image depth is 20 mm. (**c**) The B-mode CR-SO_2_ map when the rabbit is given 5% O_2_ and 95% N_2_, (**d**) 10% O_2_ and 90% N2, (**e**) 15% O_2_ and 85% N_2_, (**f**) normal air, (**g**) 30% O_2_ and 70% N_2_, and (**h**) 100% O_2_. (**i**) PAOM-CR-SO_2_ measurements [41]. CR: chorioretinal vessels. Adapted with permission from ref. [41].

A further functional capability of multimodal PAOM is the measurement of blood flow and oxygen saturation (SO_2_). Song and Liu et al. have shown that the combination of PAOM with Doppler OCT could quantify the metabolic rate of oxygen consumption. Retinal SO_2_ was estimated using spectroscopic PAM. The authors used PAM obtained at three different wavelengths of 570 nm, 578 nm, and 588 nm to identify SO_2_ in every vessels based on the molecular extinction coefficients of oxygrnated hemoglobin (HbO_2_) and deoxygenated hemoglobin (Hb) at corresponding optical wavelengths (Figure 11a) [9]. Blood flow velocity was determined by Doppler OCT (Figure 11a3,a4). Kelly-Goss et al. estimated the hemoglobin content, oxygen saturation, and blond flow in corneal neovascularization model using PAOM (Figure 11b) [85]. Hariri et al. described a high-speed multimodal photoacoustic and ultrasound imaging (PAOI) to detect chorioretinal oxygen gradient in an in vivo model of hypoxia, as well as an ischemia reperfusion model on live rabbits [41]. In this study, PA imaging was integrated with a high-frequency ultrasound system (Figure 12a). A tunable laser (680 nm to 970 nm) was applied as a light source with a pulse width of six nanoseconds, and a pulse repetition rate of 20 Hz. The laser light was delivered into the surface of the eye using optical fiber bundles. A linear array ultrasound transducer with a center frequency of 15 MHz was used to detect acouctic signals. The lateral and axial resolution of the system are 580 μm and 290 μm, respectively. Two different wavelengths of 750 nm and 850 nm were used to measure the retinal and choroidal SO_2_, as shown in Figure 12b. These wavelengths were selected due to their lower light attenuation in tissue and corresponded to the peak absorption of deoxygenated hemoglobin and oxygenated hemoglobin [90].

### 5.8. Pre-Clinical

PAOM imaging plays an important role as a non-invasive platform for the anatomical, functional, and molecular imaging of retinal diseases in both small animals, such as mice and rats, and larger animals, such as rabbits, with great potential for clinical applications. PAOM is used as a potential research tool for studying human retinal diseases such as retinal neovascularization, choroidal neovascularization, and retinal vein occlusion with high resolution and deep tissue penetration. The major biomedical imaging applications of all three kinds of PA imaging modes (PAT, AR-PAM, and OR-PAM), from multiscale structural imaging to functional imaging using endogenous biomolecules and exogenous contrast agents, have been widely studied. For example, PAT has been used to image the retinal vasculature of the rabbit eye [22]. AR-PAM has been used to study the variations in retinal blood oxygenation [41]. OR-PAM has been used to image retinal, corneal, and choroidal neovascularization, as well as melanin, in mice [27,57,91,92]. It has also been used to map blood SO_2_ concentrations and blood flow at the capillary level of the eye. Other OR-PAM studies have focused on monitoring dynamic changes in normal and abnormal retinal blood vessels in larger animals (e.g., rabbits) [39,42]. The maps of damaged choroidal vessels have also been acquired [39]. A summary of the applications of PAOM for evaluating the eye is shown in Table 4. We strongly believe that PAOM will serve as a potential tool for fundamental research and clinical practice.

Before integrating PAOM imaging into clinical settings for human use, it is imperative that research studies verify that high-contrast and high-resolution ocular images can be captured without affecting or damaging the sensitive neural tissue of the eye. Currently, there is no FDA-approved PAOM imaging system of the eye. Thus, most studies have performed a safety evaluation based on the ANSI safety limit for eyes, which permits a maximum single laser pulse energy of 160 nJ. The laser energy employed in most studies range from 40–80 nJ/pulse for the posterior imaging of the eyes, which is 50–75% lower than the ANSI safety limit. Currently, if the laser energy is lower than the ANSI safety limit, it can be regarded as safe. However, when the laser irradiates the eye, most of the energy is transmitted to the retina. Overexposure may cause retinal laser injury. The focal magnification (optical gain) of the eye also should be considered, which is approximately 100,000 times [93]. This means that an irradiance of 80 nJ entering the eye will be effectively increased to 8000 nJ when it reaches the retina. Thus, further safety evaluations should be conducted before applying this imaging technique on humans. Long-term safety studies of both the structure and functional assessment of vision should be performed in order to evaluate for evidence of cell injury, inflammation, and death following imaging.

## 6. Limitations and Future Directions

Although the combination of PAM with other imaging modalities such as OCT, SLO, and FM provide high-resolution multimodal images obtained from a single imaging system, there are several limitations. The first is that the integrated system requires different types of excitation light sources. Chao et al. used two kinds of illuminations [25]: tunable pulse laser light (400–2100 nm) for PAM, and superluminescent light for OCT. Song et al. also used two different light sources to achieve multimodal imaging [9]. Their system combined a tunable pulse dye laser and an argon laser at 488 nm. These light sources required co-alignment and synchronization to achieve multimodal imaging, in which can be time-consuming. To overcome this problem, a single excitation laser source is an alternative method that allows both PAM and OCT imaging. Recently, Liu et al. conducted simultaneous PAM and OCT using a single ultrafast laser source (pulse duration = three ns, repetition rate = 10 kHz) [50]. However, to combine with FM, the system needs to use an extra light source. Lee at al. used a near-infrared supercontinuum laser for both PAM and OCT. However, their study evaluated phantoms, so further in vivo experiments are needed. In addition, their near-infrared supercontinuum laser required high laser energy to induce the PA signal in the eye [95]. Thus, its safety is still being evaluated. Recently, pulse laser diodes have been developed as alternative laser excitation sources and applied to detect vasculature structure in the mouse ear [96,97]. The laser diode is compact and cost-effective, which is ideal to develop a portable multimodal imaging system. However, ophthalmic applications of laser diodes have still not yet been reported. Further studies are needed to evaluate the utility of laser diodes to detect retinal vasculature. Another challenge of PA imaging is the ultrasound coupling between a transducer and targeted tissue to maximize the detection of the PA signal. Water-based liquid and ultrasound gel have been widely used for acoustic coupling, which can help minimize the acoustic impedance mismatch and improve the detected acoustic signal amplitude [15,27,42,43,58]. In addition to ultrasound gel, balanced salt solution (BBS) can be used as an alternative coupling media for PA imaging. BSS is similar to water, but has a physiological pH and isotonic salt concentration to minimize irritation to the ocular surface, which is exquisitely sensitive. In addition, non-contact remote optical detection of the sound waves could be employed using optical interferometry to detect the photoacoustic signal [98-102].

The second limitation is that the acquired images obtained from a single imaging modality require further post-imaging processing to co-register and display on the same image plane. In fact, the PAM assessment of blood vasculature can be feasible only if all of the signal measurements are performed in real-time. Thus, an advanced ophthalmic PAM system still requires further investigation in order to achieve real-time imaging.

A third limitation is that the field of view (FOV) of PAM is limited by the ultrasound needle-shaped transducer. Chao et al. described how the raster-scan regions on the retina had a typical FOV of about 3 mm × 3 mm [25,50]. Hu, Liu, Song, and Jiao et al. reported a FOV of about 2 mm × 2 mm [33,36,50,57,103]. Joen and Liu et al. acquired an image area of 3 mm × 3 mm [51,92]. de la Zerda et al. archived an image area of 12 mm × 8 mm [22]. To acquire larger imaging regions with this system, several volumes need to be recorded at different positions and then overlaid, which is time-consuming and requires excellent compliance with the post-imaging processing technique. A more efficient method can be performed in real-time by using an array of ultrasound transducers to record a larger volume image.

A fourth limitation is the speed of acquisition. The current multimodal PAOM system can rapidly achieve high-resolution images in less than one minute, which is faster than the conventional PA system based on mechanical scanning methods, as shown in Table 5. The imaging speed is limited by the laser repetition rate. Chao et al. used a tunable pulse laser with the laser repetition of one kHz, resulting in the limited acquisition time of 65 s to acquire a volumetric PAM image with the resolution of 256 × 256 pixels. Another study reported by Liu et al. described a total image acquisition time of about 2.7 s using a single pulse laser with the pulse repetition rate of 10 kHz. Therefore, the acquisition time can still be improved by increasing the laser repetition rate. High imaging speed is needed to avoid possible motion artifacts, enabling the improvement of the image quality. Robinson et al. reported that the eye has a very short fixation time of approximately 500 ms [49]. This motion can cause image blurring or image disruption.

## 7. Conclusions

This review summarizes recent developments in photoacoustic microscopy for the evaluation and detection ocular structures such as choroidal and retinal vasculature, and ocular diseases including corneal neovascularization and choroidal neovascularization in both two dimensions and three dimensions. This review also discusses the spectroscopic PAM technique as a key capability of PAM to quantify the functional information of the retina such as oxygen saturation (SO_2_), blood flow, and retinal oxygen metabolic rate (rMRO2). Accurate quantification of hemoglobin concentrations is the ultimate goal of quantitative PAM spectroscopy and essential for the measurement of SO_2_ and rMRO2. Multimodal exogenous contrast agents (both organic and inorganic) and their potential application for the improved visualization of the retinal and choroidal microvasculature were discussed. Lastly, future developments and the potential for clinical translation offered by PAOM may provide great opportunities for the diagnosis of retinal diseases in the future.

## Figures and Tables

**Table 1.** Comparisons of current PA imaging modalities of the eye. NIR: near infrared, PAM: photoacoustic microscopy, PAOM: photoacoustic ophthalmoscopy.

Specification	PAT	PAOM	Vevo LAZR	REFERENCE
Axial Resolution	~15 μm	~15–37 μm	44–75 μm	[23,38-41]
Lateral Resolution	~45 μm	4.1 μm		[19,39,42,43]
Acquisition Time	>10 min	~2.7 s (2 × 2 mm^2^)–60 s (3 × 3 mm^2^)	74 s (10 mm)	[27,42,44]
Imaging Depth	~ 5 cm	~1–3 mm	~2 cm	[23]
Field of view (FOV)	30 × 30 mm	<10 × 10 mm^2^	~14–23 mm wide	[27]
Application	Anterior of the eye: whole eye tissues	Anterior and posterior of the eyes: retinal vessels, choroidal vessels, and capillaries	Anterior of the eye, SO_2_	[22,34,41]
Wavelength	532, NIR window	405–2100 nm	680–970 nm	[20,38,42]
Transducer	Single element, Linear Arrays, and planar	Single element	Linear Arrays, and planar	[16,18-21,27,38,41]
Coupling media	Ultrasound gel	Ultrasound gel, water, BSS	Ultrasound gel	[23,25,39,42]
Image acquisition Mode	PAT and US	PAM	PAT, US, and Pulse Doppler	[23,38,39,41]

**Table 2.** Lateral and axial resolution of OR-PAM.

Lateral Resolution (μm)	Axial Resolution (μm)	Frequency (MHz)	Bandwidth (MHz)	REFERENCE
5	15	75	100	Hao et al. [24]
3		50	50	Jeon et al. [51]
4.1	37	27	16	Tian et al. [25]
3.6	27.7	50	50	Kim et al. [56]

**Table 3.** Summarization of common contrast agents applied for photoacoustic microcopy and optical coherence tomography. FA: fluorescein angiography, OCT: optical coherence tomography.

Contrast Agents	Characterization	Application
Organic agents		
Indocyanine green	High penetration depth	PAM, Indocyanine green angiography (ICGA)
Evans blue	Soluble and easy clearance via recticuloendothelial system (RES)	Microvascular network imaging
Cy7 fluorophore [63]	Non-toxic, biocompatible, biodegradability, high stability ad long circulation time	OCT contrast agent, tumor imaging agents
Fluorescein sodium dye	Biocompatibility	FA
Inorganic agents		
Gold nanorod [70,80,81]	High penetration depth; relative slow tissue clearance	PAM, OCT, photothermal OCT, biomarker for molecular imaging
Gold nanoprisms [82]	Work in second near infrared window; deeper image depth	Enhanced OCT angiography

**Table 4.** Prospects of PAOM.

Retinal Disease	Advantages	Drawback	References
Choroidal Neovascularization	High resolution Moderate depth resolution Strong optical absorption	Small animals: mice and rat	[86,94]
Corneal Neovascularization	High resolution Moderate depthresolution Strong optical absorption Angiography	Small animals: mice and rats	[51,85,92]
SO_2_	Measure optical absorption of hemoglobin directly More accurate than oximetry and multi-wavelength fundus photography	Requirement of image registration, and post-image processing	[27,51,85]
Retinal oxygen metabolic rate (rMRO2)	Accurate and non-invasive quantification Derive from the measured SO_2_, blood flow and vessels diameter	Requirement of image registration	[9]
Blood flow	Accurate	Combination with OCT	[85]

**Table 5.** Summary of acquisition time of photoacoustic ophthalmic microscopy.

Scanning Method	Acquisition Time	Imaging Size	Wavelength	Energy	Application	References
Mechanical scanning	90 min	12 × 8 mm^2^	740 nm	0.5 mJ/cm^2^	Eye tissues	de la Zerda et al. [22]
Mechanical scanning	120 min	2 × 2 mm^2^	570 nm and 578 nm	40 nJ	Iris microvasculature	Hu et al. [36]
Mechanical scanning	20 min	3 × 3 mm^2^	532 nm	80 nJ	Corneal neovascularization	Liu et al. [87]
Mechanical scanning	6.5 min	2 × 2 mm^2^	532 nm	500 nJ	Iris microvasculature	Wu et al. [103]
Optical scanning	2.7 s	2 × 2 mm^2^	532 nm	40 nJ	Retinal blood vessels, and RPE	Jiao et al.[33]
Optical scanning	2.7 s	2 × 2 mm^2^	570 nm, 578 nm, and 588 nm	40 nJ	SO_2_, retinal and choroidal vessels	Song et al. [57]
Optical scanning	65 s	3 × 3 mm^2^	570 nm	80 nJ	Retinal and choroidal blood vessels in rabbit	Chao et al. [25,42]
